# The wisdom of crowds emerges in flocks of thousands of migrating songbirds

**DOI:** 10.1126/sciadv.aef3512

**Published:** 2026-06-24

**Authors:** Joe Morford, Patrick J. Lewin, Joe Wynn, Maria C. T. D. Belotti, Richard P. Mann, Christopher Krupenye, Dora Biro

**Affiliations:** ^1^ Department of Brain and Cognitive Sciences, University of Rochester, Rochester, NY, USA.; ^2^ Department of Biology, University of Oxford, Oxford, UK.; ^3^ Department of Earth, Ocean and Ecological Sciences, University of Liverpool, Liverpool, UK.; ^4^ Department of Natural Resource Ecology and Management, Oklahoma State University, Stillwater, OK, USA.; ^5^ Department of Statistics, School of Mathematics, University of Leeds, Leeds, UK.; ^6^ Department of Psychological and Brain Sciences, Johns Hopkins University, Baltimore, MD, USA.

## Abstract

While enhanced decision-making in larger groups, known as the wisdom of crowds, has been demonstrated in controlled experimental conditions, the intractability of observing large-scale wild groups has limited the availability of evidence from natural systems. Addressing this challenge, we analyze migratory trajectories of flocks of thousands of wild songbirds extracted from 23 years of weather radar data across the Great Lakes of North America. We show that the wisdom of crowds emerged: Larger flocks oriented more accurately toward their population’s migratory direction than smaller flocks. This likely emerged through the many-wrongs effect, with individual errors averaged out across flocks. Furthermore, flock size declined over time, a trend that may erode navigational performance and carry important ecological implications amid anthropogenic population fragmentation. Together, these findings reveal how the wisdom of crowds emerges in natural systems and underscore its role in shaping movement and ecological resilience in social animals.

## Teaser

Larger flocks of migrating songbirds orient more accurately toward their population’s migratory direction than smaller flocks.

## INTRODUCTION

More than two millennia ago, Aristotle proposed that collective decision-making could surpass the performance of even the best individual members of a group, a notion now popularized as the wisdom of crowds, collective intelligence, or swarm intelligence (*1*). While he was more equivocal about the idea that crowds of “wild beasts” could achieve the same (*2*), growing empirical evidence demonstrates that, in certain contexts, larger animal groups can outperform smaller groups and individuals (*3*–*8*). However, as most studies have focused on decision-making in captive animals under controlled experimental conditions, direct evidence from large groups in the wild remains rare, with few exceptions (*9*). One domain where the wisdom of crowds has been widely theorized to emerge is navigation, whereby individual directional errors could average out across groups, allowing larger groups to orient more accurately than smaller groups, an idea termed the “many-wrongs” effect (*3*, *10*–*16*). However, empirical support in natural systems is restricted to a small number of experimental studies in domesticated homing pigeons (*3*, *17*, *18*). Therefore, how navigational accuracy scales with group size in natural conditions remains poorly understood. Here, we show that the wisdom of crowds emerges in flocks of thousands of migratory swallows and martins, most likely through the many-wrongs effect, with larger flocks oriented more accurately toward their population’s migratory direction than smaller flocks.

Every year, billions of animals perform long-distance migrations; while some migrants are entirely solitary, many migrate in groups varying in size and stability across taxa. Social migration opens the possibility for emergent group-level phenomena (*19*–*23*), including both cross-generational cultural transmission of travel routes and improved navigational accuracy through the many-wrongs effect. Such effects could, in principle, have important consequences for the viability of migratory animals as populations fragment in the Anthropocene (*24*). It has been suggested that the navigational and foraging efficiency of passenger pigeon flocks (*Ectopistes migratorius*) dropped as their population declined because of excessive hunting (*25*), playing a role in driving their extinction in the early 20th century, an example of the Allee effect (*26*). Yet, whether these effects reliably emerge in migrating animal groups and what ecological consequences they hold for social animals remain open questions.

We used a large dataset of departure directions of hirundine flocks from overnight roosts, derived from 12 weather surveillance radar stations in the Great Lakes region of the United States over 23 years (*27*). Hirundines, including purple martins (*Progne subis*), tree swallows (*Tachycineta bicolor*), barn swallows (*Hirundo rustica*), cliff swallows (*Petrochelidon pyrrhonota*), and bank swallows (*Riparia riparia*), roost overnight in this region in large flocks. Roosts are typically dominated by a single species and occur during the postbreeding season from late July to early October, before and during their southward migration. On migration, these birds travel several hundred kilometers each day (*28*, *29*); these movements are likely guided by compass orientation, although the specific cues and mechanisms underlying this behavior remain poorly understood (*30*). Between dawn and sunrise, these birds synchronously depart their roosts, generating characteristic ring-shaped traces in weather radar as they move and disperse (*31*–*33*). These signature traces enable the use of machine learning approaches to automate their detection (*27*, *34*, *35*), facilitating long-term and large-scale quantification and analysis of population trends and examination of their behavior. Crucially, detecting traces of a single flock in successive radar scans enables the reconstruction of the flocks’ trajectory from its roost, allowing us to determine its migratory departure direction (direction from first to last detection).

## RESULTS AND DISCUSSION

### Radar detection of bird flocks on migration

**Fig. 1.**
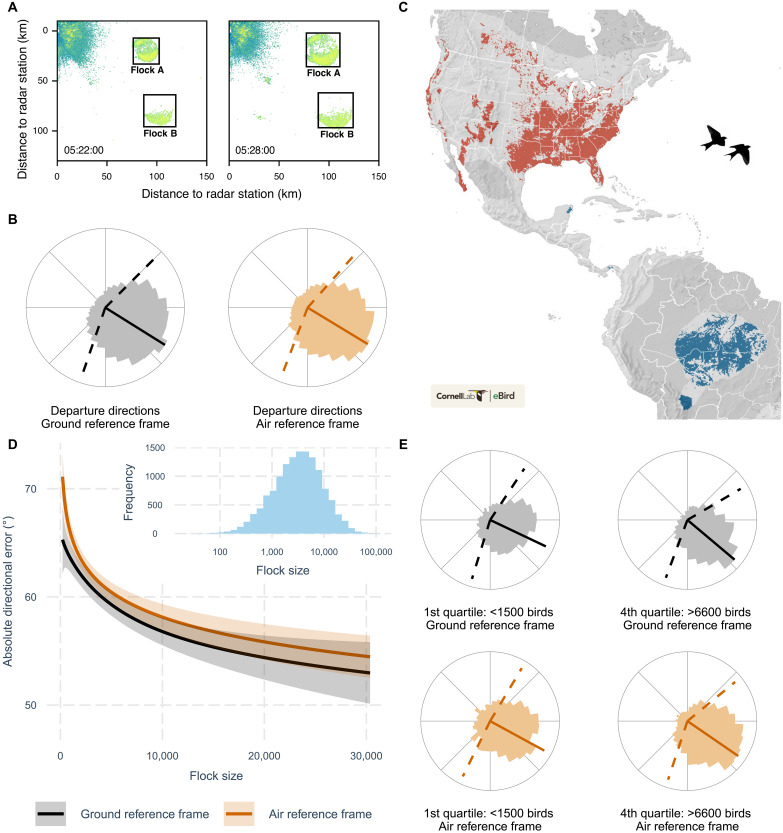
The wisdom of crowds emerges in migratory departure directions. (**A**) Flocks, detected in successive radar traces from the KDTX station on 12 August 2016, are shown. Brighter coloration signifies higher reflectivity and, hence, higher biomass density. Local time is reported in each trace. (**B**) Departure directions of flocks leaving their roosts are shown in circular histograms. The circular mean directions and the means ± circular SDs are shown by solid and dashed lines. (**C**) A range map of purple martins, adapted from eBird Status and Trends (*36*), is shown for breeding in red and nonbreeding in blue. Dark gray indicates regions with no predictions due to insufficient data. Range map derived from abundance data provided by Cornell Lab of Ornithology | eBird (https://science.ebird.org/en/status-and-trends). (**D**) Generalized additive models (GAMs) show absolute directional error decreases with increasing flock size (displayed across 1st to 99th percentiles of flock size). Smooths and 95% confidence intervals are included. The inset shows the distribution of estimated flock sizes. (**E**) Circular histograms of flock orientations for the smallest (left) and largest (right) quartiles of flock size are shown. The circular mean directions are marked by a solid line and the means ± circular SD by dashed lines. Approximate flock size ranges for each quartile are shown below.

From a sample of 13,577 flock trajectories, from 57,201 individual flock detections (see examples in Fig. 1A), we found that flocks were oriented, on average, in a south-easterly direction with a circular mean of 122.0° and a circular SD of 76.9° (Fig. 1B). To determine the extent to which the directional distribution might be influenced by local wind conditions, we tested for correlations between the east-west wind velocity and east-west movement of flocks, and likewise between the north-south wind velocity and north-south movements. In each case, we found strong positive correlations (east-west: Spearman’s ρ = 0.41, *P* < 0.0001; north-south: ρ = 0.48, *P* < 0.0001), indicating that wind conditions influence the directional movements of the birds. To account for this, we recalculated the movement of the birds in the air reference frame, through vector subtraction of the wind vector from the movements of the flocks. In the air reference frame, we again found that the flocks were oriented in a south-easterly direction (Fig. 1B; circular mean: 121.2°; circular SD: 79.6°), but that the positive correlations between wind velocities and flock movements were abolished (east-west: ρ = −0.0026, *P* = 0.759; north-south: ρ = −0.028, *P* = 0.0009). This suggests that this is a reasonable correction for the influence of the wind conditions on the movement of the flocks and demonstrates that our distribution of departure directions is not merely an artifact of prevailing wind directions. These departure directions are consistent with the direction between the breeding and nonbreeding grounds of these species [see purple martin range map, Fig. 1C, adapted from eBird Status and Trends (*36*); range map derived from abundance data provided by Cornell Lab of Ornithology | eBird, https://science.ebird.org/en/status-and-trends]. Moreover, the observed variation in departure directions is substantially greater than the variation in migratory bearing required to travel from breeding to nonbreeding sites across the ranges of each species. This disparity implies that much of the spread reflects inaccuracy in migratory orientation; under such conditions, averaging across individuals might be expected to cancel random errors and yield more accurate orientation in larger flocks.

The number of individuals in each flock can be estimated using the reflectivity and spatial size of radar traces. Like a previous study (*27*), we estimated flock sizes conservatively by assuming that flocks comprise the largest of the five represented hirundine species, the purple martin, and, to obtain a flock size estimate across the trajectory of each flock, we took the mean estimate across traces. Estimated flock sizes varied between ~100 and 100,000, but most flocks comprised between 1000 and 10,000 individual birds (Fig. 1D, inset).

### The emergence of the wisdom of crowds

To test for the emergence of the wisdom of crowds, we examined whether larger flocks navigated more accurately than smaller flocks. Under the many-wrongs effect, we expected a negative relationship between directional error and flock size and that this relationship would be steepest at smaller flock sizes, then plateauing with increasing flock size, conferring diminishing returns in accuracy improvements as flocks grow larger (*37*). Therefore, first, we tested for a negative correlation between flock size and absolute directional error from the population circular mean direction. We found a significant negative correlation between directional error and flock size (Spearman’s ρ = −0.067, *n* = 13,577, *P* < 0.0001), providing evidence that larger flocks were better oriented than smaller flocks in the population migratory direction. This result replicated for directional errors in the air reference frame (corrected for wind), again with a significant negative correlation between error and flock size (ρ = −0.073, *n* = 13,577, *P* < 0.0001). To further characterize the shape of this relationship and assess its robustness, we fitted generalized additive models (GAMs) to model absolute directional error as a function of log-transformed flock size. The models revealed a significant smooth term in both reference frames [ground, uncorrected for wind: effective degrees of freedom (edf) = 2.30, *F* = 20.3, *P* < 0.0001; air: edf = 1.06, *F* = 73.2, *P* < 0.0001], supporting a nonlinear but consistently negative relationship between flock size and directional error. As shown in Fig. 1D, the relationship was steepest at smaller group sizes, indicating diminishing returns in accuracy improvements as flocks grow larger, as expected under the many-wrongs effect. The decrease in navigational error with group size is captured by the circular SD falling from 81.7° (or 87.7° in air reference frame) in the smallest quartile of flocks to 70.4° (air: 73.8°) in the largest quartile (Fig. 1E). Hence, these results are consistent with the predictions of the many-wrongs effect, in which the integration of multiple individual estimates can improve collective accuracy through averaging, although alternative mechanisms cannot be definitively ruled out. Averaging across a group could be produced by local interactions between neighboring birds (*10*, *24*, *38*, *39*), rather than requiring global communication or control, and hence is a plausible mechanism for facilitating the emergence of the wisdom of crowds in this system.

To verify that this result was not an artifact of possible confounding factors, including species composition, seasonal changes in migratory behavior, or errors in flock position estimates, we conducted a series of follow-up analyses. First, to establish whether this result could be driven by seasonal changes in flock sizes, perhaps in association with changes in migratory tendencies, we examined these relationships after accounting for seasonal changes in average migratory direction. We determined how the average migratory direction changed through the season by taking a smoothed mean direction of flock orientations by Julian date (see Materials and Methods) and calculated directional errors of the trajectory of each flock from the daily circular mean direction. Again, we found significant negative correlations between directional errors and flock size, in both ground (ρ = −0.056, *n* = 13,291, *P* < 0.0001) and air (ρ = −0.069, *n* = 13,291, *P* < 0.0001) reference frames. Additionally, we fitted GAMs, revealing significant smooth terms in both reference frames (ground: edf = 2.22, *F* = 13.9, *P* < 0.0001; air: edf = 1.06, *F* = 61.9, *P* < 0.0001) and confirming that the negative, nonlinear relationship between flock size and directional error persisted after accounting for seasonal changes in orientation.

Next, we examined whether the negative relationship between directional error and group size could be explained by the orientation of larger flocks being better estimated. For instance, if larger flocks were detected for longer distances, then this could generate more accurate estimates of the orientations of larger than smaller flocks. To rule this out, we confirmed that measuring departure direction as the bearing between the first and second detections of flocks (rather than between the first and last) generated no substantive changes in results (flock size and error correlation in ground reference frame: ρ *=* −0.048, *n* = 13,577, *P* < 0.0001; air: ρ *=* −0.081, *n* = 13,577, *P* < 0.0001). Further, if the positions of larger flocks were better estimated than smaller flocks, then we might expect a greater spread of directional estimates within the trajectories of smaller flocks. However, we found no evidence for this, instead finding that within-track circular SD increased with flock size (Spearman’s ρ *=* 0.244, *P* < 0.0001). Last, we found that our results were robust to the use of different methods for measuring flock size: No substantive changes in results were found when maximum (rather than mean) estimated flocks size across a flock’s trajectory was examined (flock size and error correlation in ground reference frame: ρ *=* −0.069, *n* = 13,577, *P* < 0.0001; air: ρ *=* −0.072, *n* = 13,577, *P* < 0.0001). Together, these results indicate that the observed relationship between flock size and directional accuracy is unlikely to be an artifact of measurement bias.

### Flocks confirmed as swallows and martins

**Fig. 2.**
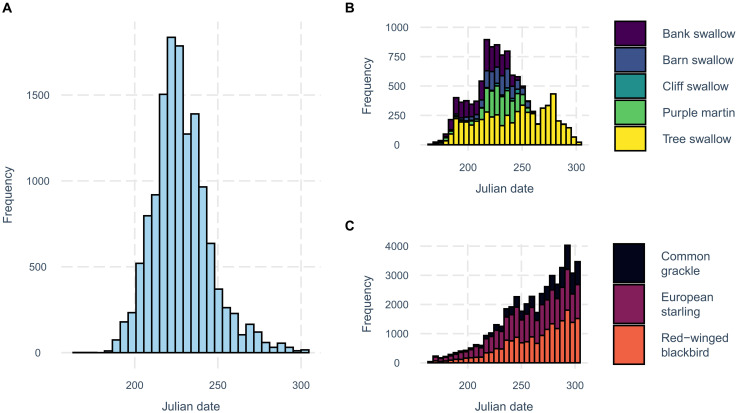
Seasonal timing of detections consistent with hirundine phenology. (**A**) Histogram showing the seasonal timing of radar detections of flocks. These detections are consistent with flocks comprising hirundine species. (**B**) Stacked histogram of eBird observations of large hirundine flocks (>200 individuals), supporting the interpretation of radar data in (A). From these observations, we infer that radar detections later in the season are likely dominated by tree swallow flocks. (**C**) Stacked histogram of eBird observations of other communally roosting species in this region shows that their phenology is inconsistent with the radar detections in (A).

We sought to confirm that the detected flocks were predominantly composed of the five hirundine species identified in previous studies and to rule out the possibility that the results were driven by confounds related to species composition. To verify the species composition, we cross-referenced our radar detections with direct observations of large bird flocks from eBird (*40*), a global citizen science database maintained by the Cornell Lab of Ornithology (eBird Basic Dataset. Version: EBD_relJan-2025. Cornell Lab of Ornithology, Ithaca, New York. January 2025), in the same geographical area over the same time period. In concordance with previous studies (*27*, *31*–*33*), we found that seasonal timings of the radar detections were consistent with direct observations of these hirundine species (Fig. 2, A and B). Conversely, the seasonal timings of radar detections were inconsistent with observations of other species that flock in large numbers in this region (Fig. 2C), including common starlings (*Sturnus vulgaris*), red-winged blackbirds (*Agelaius phoeniceus*), and common grackles (*Quiscalus quiscula*): The number of sightings of large flocks of these species increased through October as radar detections approached zero. This indicates that these species were little represented in the radar detections of bird flocks, likely because their departure from roosting sites generates radar traces that are distinct from the characteristic ring-shaped traces of the swallows and martins.

We found that tree swallows were present in the region later than any of the other hirundine species, accounting for more than 95% of observed flocks on any given date after Julian date 260 (mid-September). This indicates that the flock detections later in the season were likely to be dominated by tree swallows, enabling us to analyze a portion of the dataset dominated by this single species (number of flocks detected after Julian date 260 = 674). We found evidence for the wisdom of the crowds in this portion of the dataset as well, with the negative correlations between directional error and group size replicated despite a much reduced sample size (ground reference frame: ρ *=* −0.078, *n* = 674, *P* = 0.042; air reference frame: ρ *=* −0.10, *n* = 674, *P* = 0.0098), ruling out the possibility that the negative correlations across the whole dataset could be explained merely by an artifact related to variation in species composition.

### Environmental biases and collective intelligence

The signature of the many-wrongs effect shown above indicates that the wisdom of crowds likely emerged here through flocks integrating individual estimates of migratory direction. Local interactions among neighboring birds could scale up to produce an averaging effect (*10*, *38*), provided that individual directional errors are at least partly independent, rather than representing biases from the population migratory direction common to all members of the flock. However, shared environmental factors, such as wind, may introduce biases—correlated errors—within flocks, limiting the benefits of averaging and thus weakening any collective intelligence effect. We therefore expected the strength of this effect to depend on the balance between uncorrelated and correlated errors among individuals. To test the influence of wind as a source of correlated error, we compared flock trajectories in the ground reference frame (uncorrected for wind) and the air frame (corrected for wind). If wind induced correlated errors, then we should expect a stronger correlation between flock size and directional error in the air frame. As predicted, the correlation coefficient was stronger in the air frame (ground: ρ = −0.067; air: ρ = −0.073); however, bootstrapping showed these differences were not statistically significant (*P* = 0.342, number of bootstrap iterations = 1000). Therefore, we found no strong evidence that wind-induced correlated errors reduced the strength of collective intelligence, and so the role of environmental factors in shaping collective intelligence remains to be seen.

### Ecological and conservation implications

**Fig. 3.**
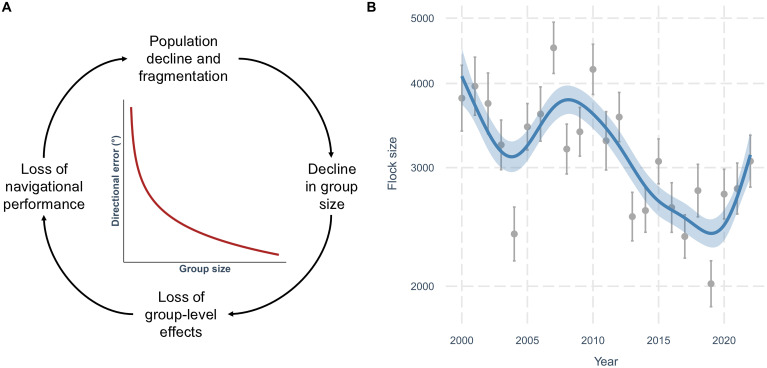
Group size declines. (**A**) A potential feedback loop between population declines and reductions in navigational performance. This could be exacerbated by the nonlinear relationship between navigational accuracy and flock size, generating an accelerating decline in navigational performance. (**B**) A GAM reveals the nonlinear relationship between year and log-transformed flock size, with an overall decline in average flock size over the 23-year period. The shaded region indicates the 95% confidence interval of the smooth term. The geometric mean flock size and confidence intervals are shown for each year in gray.

The emergence of the wisdom of crowds in this study raises the possibility that year-on-year changes in flock sizes could influence the navigational performance of flocks, with potential downstream ecological consequences. Moreover, this suggests the risk of a positive feedback loop: If population declines and fragmentation lead to decreases in flock size (*41*), then navigational performance may deteriorate (*24*), further exacerbating population loss. This would be intensified by the nonlinear relationship between flock size and navigational accuracy generated by the many-wrongs effect, potentially driving an accelerating decline in navigational performance (Fig. 3A).

Across the study period, flock sizes declined, with a significant negative correlation between year and geometric mean flock size (Spearman’s ρ *=* −0.625, *n* = 23, *P* = 0.0018). This replicated in the portion of the data dominated by tree swallows (after Julian date 260: ρ *=* −0.434, *n* = 23, *P* = 0.040), indicating that changes in species composition do not explain the observed decline. Fitting a GAM to log-transformed flock size revealed a nonmonotonic relationship with year (edf = 7.54, *F* = 35.1, *P* < 0.0001), showing that flock sizes have fluctuated in more complex ways over time. Nevertheless, average flock sizes were lower at the end of the study than the beginning (Fig. 3B).

We examined how changes in flock size could explain trends in navigational error over time. Using the empirically modeled relationship between flock size and navigational error, together with the annual distributions of flock size, we generated predictions for the distribution of absolute directional error for each year in both the ground and air reference frames. Fitting GAMs to examine how these predicted values varied with year revealed the expected temporal changes in absolute directional error (ground: edf = 7.69, *F* = 37.8, *P* < 0.0001; air: edf = 7.53, *F* = 35.0, *P* < 0.0001). However, these predicted changes were modest, amounting to less than 2° across the study period (maximum difference in fitted values; ground: 1.50°; air: 1.80°), and, therefore, too subtle to be detected in the observed data. Consequently, we did not attempt to directly test for variation in navigational error driven by year-to-year changes in flock size.

This limited predicted effect reflects the nonlinear relationship between flock size and navigational error: At the flock sizes observed during the study period, reductions in flock size have only a modest impact on error. However, further decreases in flock size would shift flocks into a range where performance deteriorates more rapidly, potentially causing an accelerating increase in navigational error. Therefore, although we found no evidence that temporal declines in flock size drove changes in migratory performance during the study, we suggest that the potential loss of the wisdom of crowds may have ecological implications as populations decline. To better evaluate these predictions, future work could combine longer-term and broader-scale observational datasets to capture sufficient variation in flock sizes over time, alongside modeling to predict the magnitude of such effects. These approaches would help to ground this discussion and provide clearer directions for investigating the consequences of declining group sizes in wild populations.

In conclusion, this study provides evidence that the wisdom of crowds emerges in large-scale natural systems, reflected in substantial improvements in migratory directions across flocks of thousands of birds. We found that larger flocks more accurately oriented toward their population’s migratory direction that smaller flocks and that this relationship was steepest at smaller flock sizes, indicating diminishing returns in accuracy as flocks become larger. This is consistent with the many-wrongs effect, whereby individual directional errors are averaged out across group members, thus supporting experimental findings with small flocks of homing pigeons (*3*) and extending this principle to large-scale wild migratory systems. Future work could examine whether these findings extend across other migratory taxa and quantify the scale of the navigational benefits that it confers in diverse natural systems.

Last, we document a decline in flock sizes over time within the study region, raising the possibility that continued reductions could impair navigational performance. Further, our demonstration that navigational performance depends nonlinearly on flock size suggests that apparent resilience in the face of population decline could rapidly erode if flock sizes fall below critical values. This raises the dangerous possibility that deteriorations in performance might not be observed until a sudden collapse. These findings highlight the broader ecological and conservation implications of group-level phenomena like the wisdom of crowds, particularly in the context of ongoing anthropogenic population fragmentation and decline (*23*, *42*). To evaluate these implications further, future research should explore how population density and group size interact across taxa, characterize how group-level effects emerge and scale in natural systems, and evaluate their consequences for the persistence and resilience of declining wild populations.

## MATERIALS AND METHODS

The process of generating the dataset used in this study, through detecting bird flocks in weather radar data, is described in detail in a previous paper (*27*). The dataset comprises 13,577 tracks of flocks leaving their overnight roosts from 57,201 individual flock detections. Flock sizes were estimated by converting the reflectivity measurements to bird counts within each frame, assuming that birds had the average mass of an adult purple martin (the largest present species) to obtain the most conservative estimate. In the present study, these bird counts were summarized across tracks by taking the mean flock size across the frames in which the flock was detected. We calculated the departure direction of the flocks as the bearing between the first and last centroids of the detected flock positions.

To extract surface wind data, we used the Copernicus Climate Data Store to access the European Centre for Medium-Range Weather Forecasts (ECMWF) Reanalysis 5th Generation Land (ERA5-Land) dataset. This dataset provides 10-m *u*- and *v*-wind components for every 0.1° longitude and latitude in the study region. Although birds are likely flying at higher altitudes during roost departure, surface-level wind data serve as a reasonable proxy for flight conditions during roost departure. We extracted these wind components for the closest coordinate at the nearest hour for each flock detection in the weather radar data. We examined the correlation between the *u*- and *v*-wind velocities and the movements of flocks in these two axes, using the nonparametric Spearman’s rank correlation test. Subsequently, using vector subtraction of the movement vectors of the air from the movement vectors of the flocks from the first to last detection of each flock, we calculated the movement of the birds in the air frame of reference. We then examined the correlations between the *u*- and *v*-wind velocities and the movements of flocks in the air frame of reference in the *u* and *v* axes to assess how well the vector subtraction corrects for the influence of wind of the movements of the birds.

To test for the emergence of the wisdom of crowds, we used nonparametric Spearman’s rank correlation tests between estimated flock size and the absolute directional error from the population circular mean direction, which assumes a monotonic but not necessarily linear relationship between variables. This was implemented in both the ground and air reference frames. Additionally, to account for any seasonal changes in flock sizes and the distribution of migratory directions, we examined these relationships after accounting for seasonal changes in average migratory direction. To account for seasonal changes, we took a smoothed mean direction of flock orientations by Julian date, applying a ±14-day window around each unique Julian date. To avoid edge effects, only data between the 1st and 99th percentiles of Julian dates were included in the analysis.

To characterize the relationship between flock size and navigational accuracy, we fitted GAMs with a Gaussian error structure and estimated smoothing parameters using restricted maximum likelihood (REML). Absolute directional error (in both ground and air reference frames) was modeled as a smooth function of log-transformed flock size. This approach allowed for nonlinear relationships and tested whether improvements in navigational accuracy with increasing flock size followed the diminishing returns predicted under the many-wrongs hypothesis.

To verify the species composition of radar-detected flocks, we cross-referenced detections with direct observation records from eBird (*40*), a global citizen science database maintained by the Cornell Lab of Ornithology (eBird Basic Dataset. Version: EBD_relJan-2025. Cornell Lab of Ornithology, Ithaca, New York. January 2025). We extracted eBird observations of eight species during the study period (January 2000 to January 2025) within a 150-km radius of a focal radar station, corresponding to the approximate detection range. These species included the five hirundine species previously identified in the literature as responsible for generating such radar traces: purple martins (*P. subis*), tree swallows (*T. bicolor*), barn swallows (*H. rustica*), cliff swallows (*P. pyrrhonota*), and bank swallows (*R. riparia*). Additionally, three other species that roost in large numbers in this region were included: common starlings (*S. vulgaris*), red-winged blackbirds (*A. phoeniceus*), and common grackles (*Q. quiscula*). We filtered the dataset to include only observations of flocks estimated by observers to contain more than 200 individuals, consistent with the size distribution of radar-detected flocks (more than 99% exceeded 200 individuals). We compared the seasonal timings of radar detected flocks and these citizen science observations to determine which species were likely to be contributing to our dataset, finding, in concordance with previous studies, that the radar detections likely comprised hirundine flocks in most cases. Further, we found that tree swallows are present in this region later than any of the other four hirundine species, with this species comprising more than 95% of observed flocks of these five species on any given date after Julian date 260. This allowed us to repeat the analyses above in a portion of the dataset dominated by a single species (number of flocks detected after Julian date 260 = 674) to determine whether we could rule out that an artifact of the species composition was driving the main results.

To evaluate whether wind-induced correlated errors reduce the strength of collective decision-making in migratory flocks, we tested for differences in the relationship between flock size and directional error using two reference frames: ground (unadjusted for wind) and air (corrected for wind). We predicted that the negative correlation between flock size and directional error would be stronger in the air reference frame, where wind influence is removed. We used a bootstrapping procedure to compare the two correlation coefficients by resampling tracks from the dataset with replacement for a total of 1000 iterations. In each iteration, we calculated both correlation coefficients using the same sample and computed the proportion of iterations in which the ground reference frame showed a more negative correlation than the air reference frame. Twice this proportion was taken as a two-tailed *P* value assessing the significance of the difference.

To assess long-term changes in flock size and their potential effects on navigational performance, we tested for a relationship between year and estimated flock size across the dataset. We computed the geometric mean flock size per year and tested for monotonic trends using Spearman’s rank correlation. To account for possible nonlinear patterns, we also fitted a GAM, with REML, to log-transformed flock size as a function of year. To examine how these temporal changes in flock size could explain trends in navigational error over time, we used the empirically modeled relationship between flock size and navigational error (from GAMs between absolute directional error and log-transformed flock size in both ground and air reference frames) to generate predictions for the distribution of absolute directional error for each year from the annual distributions of flock size (in each of the ground and air reference frames). We then fitted GAMs to examine how these predicted values of navigational error varied with year. Last, we calculated the total range of predicted temporal variation in navigational error by calculating the difference between the minimum and maximum fitted values of the GAMs across the study period (separately for each reference frame).
